# Solving the controversy of healthier organic fruit: Leaf wounding triggers distant gene expression response of polyphenol biosynthesis in strawberry fruit (*Fragaria x ananassa*)

**DOI:** 10.1038/s41598-019-55033-w

**Published:** 2019-12-17

**Authors:** Facundo Ibanez, Woo Young Bang, Leonardo Lombardini, Luis Cisneros-Zevallos

**Affiliations:** 1 Molecular and Environmental Plant Sciences Program, Texas A&M University, College Station, Texas, USA; 2 Department of Horticultural Sciences, Texas A&M University, College Station, Texas, USA; 3 Instituto Nacional de Investigación Agropecuaria (INIA), Estación Experimental INIA Las Brujas, Canelones, Uruguay

**Keywords:** Wounding, Biosynthesis

## Abstract

The claim that organic agriculture produces higher levels of phytochemicals has been controversial for decades. Using strawberries as a model crop in field conditions, a preharvest leaf wounding stress was applied to study the production of phytochemicals in fruits. As a result phenolic compounds (PCs) and total soluble sugars increased significantly, where specific phenylpropanoids showed increment up to 137% and several genes related to PCs biosynthesis and sugar transport were overexpressed. It was observed that the accumulation of PCs on fruits can be triggered by the application of wounding stress in a distant tissue and this accumulation is directly related to carbon partition and associated gene expression. This supports the idea that higher levels of healthy phytochemicals reported in organic fruits and vegetables could be due to the wounding component of the biotic stress attributed to insects to which the plant are exposed to.

## Introduction

The organic food market have grown between 17% and 21% in the last years, compared to the 2–4% growth for conventional food products market^1^. European countries and United States lead the global market as producers and consumers, and several other countries, including Australia, China, Argentina, Brazil and Uruguay are important producers for export markets^2^.

It has been supported by several studies that organic fruit and vegetables contain higher levels of secondary metabolites related to plant defenses^3–5^. Meta-analysis showed that the content of secondary metabolites in organic products was 12% higher than in those grown with conventional practices^4^. In general, vegetables and fruits from organic production contain greater amounts of anthocyanins, flavonoids and carotenoids^5^. Higher levels of phytochemicals, particularly phenolic compounds, could be related to higher levels of biotic stress, such as insect damage, when plants are grown in organic conditions^6^. Stresses like wounding and those induced by herbivores (biotic stress) cause changes in plant secondary metabolism^7^. Wounded tissues alter the production of phenylpropanoid secondary metabolites as a local response, and also as a systemic response in the same organ type (e.g. leaves)^8^. Plants under attack from herbivores develop an efficient defense system that involves a crosstalk between signaling molecules including phytohormones such as salicylic acid (SA), jasmonic acid (JA), ethylene (ET), abscisic acid (ABA), indoleacetic acid (IAA), gibberellic acid (GA), and several reactive oxygen species (ROSs)^9,10^. The ROSs has critical roles in signaling related to plant defenses, among several other functions^11,12^. Specific elicitors released by the insect activate several signaling pathways that interact with each other (crosstalk) producing a metabolic rearrangement, expressing defense related genes and sometimes directly releasing volatile organic compounds^13^. Jasmonic acid, methyl jasmonate (MeJA) and its precursor 12-oxo-phytodienoic acid (OPDA) are inducers of proteinase inhibitors as a main defense against herbivore feeding^14^. Jasmonic acid and ET play an important role as positive regulators of plant defense against insect attack and some pathogens, whereas SA has been associated with resistance against pathogens^15^. The crosstalk allows the plant to optimize responses against herbivores and pathogens, and this strategy produces a very complex defensive system^9,11^.

**Figure 1:**
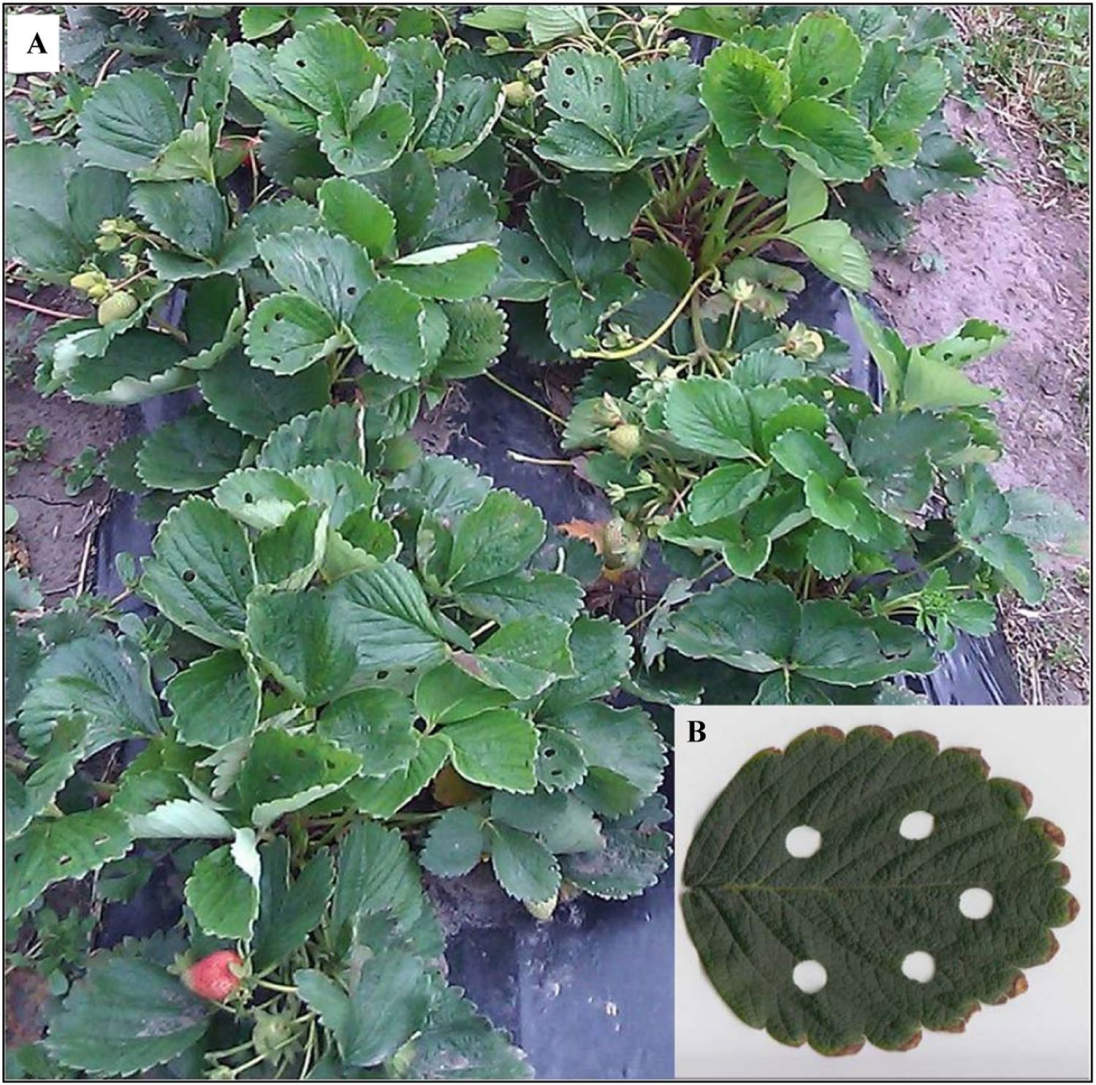
Picture of strawberry wounding experiment with 50 perforations per plant (W50). (**A**) Field experiment. (**B**) sample leaf with mechanical wounding.

The importance of plant defensive compounds (phytochemicals) for human health has led to the study of pre- and post-harvest factors that influence the production of bioactive phenylpropanoids^6,16^. Phenylpropanoids and ellagitannins received attention for their biological activity associated with human health benefits like antioxidant, anti-allergy, anti-hypertensive, antitumor effects *in vitro* and *in vivo*^17,18^. Studies evaluating secondary metabolite responses to biotic stress have shown an induction of phenolic compounds production and other phytoalexins as a local and systemic plant defensive response; however, these studies were conducted in the same tissue where the damage had been caused by piercing-sucking insects^9,19^ or leaf chewing insects^20,21^. In the present study we evaluated the systemic induction of secondary metabolites in fruits when the stress is applied in a distant organ of the plant (e.g., leaves, Fig. 1) and proposed a mechanism of phenylpropanoid accumulation in the fruit and how it is directly related to carbon partition and associated gene expression.

## Results

### Wounding stress on leaves and phenolic biosynthesis in fruit

**Figure 2:**
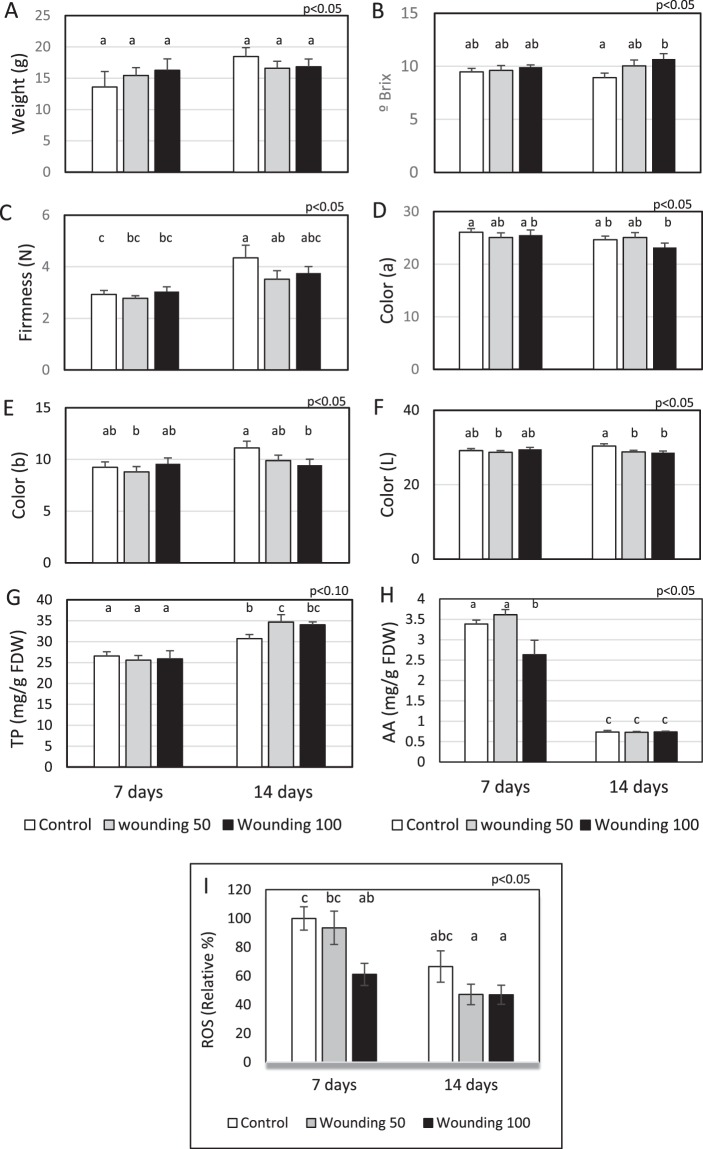
Application of two levels of preharvest wounding on leaves and its effects in strawberry fruit when wounding occurred 7 and 14 days before harvest. Each bar represent the average ± SE of individual fresh fruit evaluated from 15–20 plants; weight (**A**); soluble sugars (**B**); firmness (**C**); color (L a b system, **D**–**F** respectively); total phenolics, TP (**G**); ascorbic acid, AA (**H**) and reactive oxygen species, ROS (**I**). TP and AA were measured in freeze-dried fruits and expressed per g of freeze-dried weight (FDW). In each test different letters indicate a significant difference among treatments and controls (Duncan’s test, *p* ≤ 0.05, *p* ≤ 0.10).

The evaluation of several fruit quality parameters was carried out immediately after harvest. Average fruit weight (Fig. 2A), firmness (Fig. 2C), and color (Fig. 2D–F), at harvest was the same after 7 and 14 days of applied wounding. For soluble solids, meaning soluble sugars and organic acids, a significant increment (19.7%) in fruit was observed in W100 treated plants (100 perforations per plant) when wounding occurred on leaves two weeks before harvest (Fig. 2B). Moreover, the amount of total phenolics (TP) in fruits of all treated plants increased significantly, 12.8% and 10.7% over the control when wounding ocurred 1 and 2 weeks, respectively, before harvest (Fig. 2G). Overall total ascorbic acid decreased with time (Fig. 2H). Total ascorbic acid was significantly less for the higher level of wounding (W100) applied 7 days before harvest but the difference was not apparent when wounding occurred 14 days before harvest. In addition, physiological levels of reactive oxygen species (ROS) showed similar trend as vitamin C (Fig. 2I).

A significant increase in the level of specific phenylpropanoids and tannins derivatives in fruit was observed for W100 treated plants when wounding occurred 2 weeks before harvest compared with the control including ellagic acid (+58%), epicatechin (+100%), gallic acid (+68%), quercetin (+190%), and rutin (+137%) (Table 1).

**Table 1:** Ellagic acid, epicatechin, gallic acid, quercetin and rutin content in strawberry fruit evaluated after application of two levels of pre-harvest wounding on leaves 7 and 14 days before harvest.

Wounding before Harvest (time)	Treatment	Ellagic acid^a,b,c^ (µg/g FDW)^d^	Epicatechin^a,b^ (µg/g FDW)^d^	Gallic acid^a,b^ (µg/g FDW)^d^	Quercetin^a,b^ (µg/g FDW)^d^	Rutin^a,b^ (µg/g FDW)^d^
7 days	Control	0.21 ± 0.06 a	0.56 ± 0.16 a	2.55 ± 0.60 a	0.29 ± 0.25 a	0.27 ± 0.09 a
	Wounding 50	0.78 ± 0.42 a	1.39 ± 0.93 a	3.26 ± 1.38 a	0.05 ± 0.02 a	0.24 ± 0.01 a
	Wounding 100	0.35 ± 0.30 a	0.31 ± 0.19 a	0.64 ± 0.38 a	0.05 ± 0.01 a	0.26 ± 0.13 a
14 days	Control	178.75 ± 52.21 b	37.28 ± 5.01 b	380.20 ± 48.78 b	4.26 ± 0.63 ab	35.30 ± 6.18 a
	Wounding 50	211.77 ± 23.90 bc	97.19 ± 6.78 c	905.51 ± 69.67 c	8.84 ± 1.26 ab	102.54 ± 17.67 b
	Wounding 100	283.17 ± 50.73 c	74.37 ± 16.44 c	628.82 ± 62.16 d	12.39 ± 6.10 b	83.80 ± 21.75 b

^a^Data expressed as means ± SE. Identification of each compound (Retention time, UV λ max, [M-H]- m/z, MS fragments) was done according to the following standards: Ellagic acid (17,19 min; 255,368 nm; 301 m/z; **163**, 135), Epicatechin (16.6 min; 236, 279 nm; 289 m/z; **163**, 159, 145, 137); Gallic acid (12.60 min; 227, 272 nm; 169 m/z; **125**, 81); Quercetin (22.10 min; 256, 371 nm; 301 m/z; 283, 227, **163**, 149); Rutin (17.23 min; 256, 355 nm; 609 m/z; 463, 447, **301**, 255).

^b^Means with a common letter in the same column are not significantly different at *p* ≤ 0.05 or *p* ≤ 0.10 for ellagic acid^c^ (Duncan’s Test).

^d^(µg/g FDW): micrograms of compound per g of freeze dried weight of strawberry fruit.

### Wounding stress on leaves and gene response in fruit

**Figure 3:**
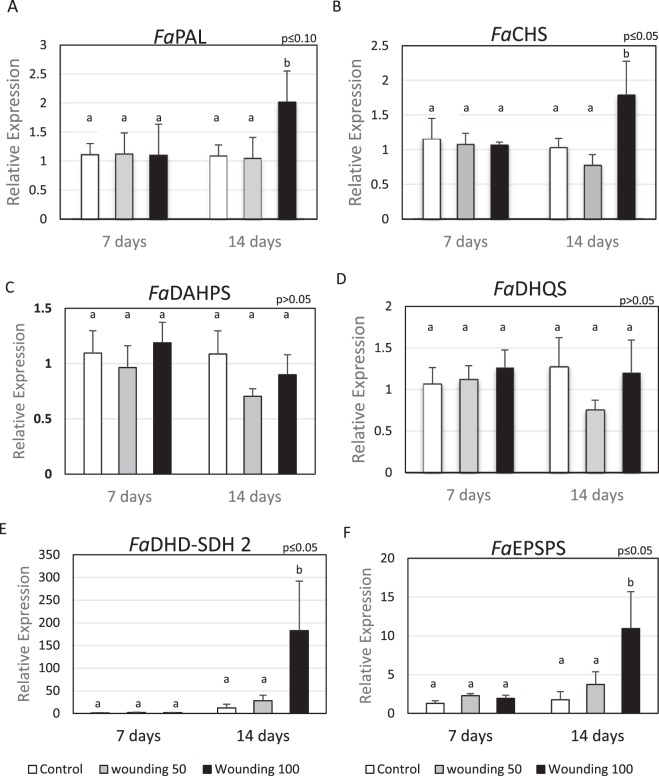
Relative expression of phenylpropanoid intermediates genes. Application of two levels of preharvest wounding on leaves and its effects in strawberry fruit when wounding occurred 7 and 14 days before harvest. (**A**) Phenylalanine ammonia lyase (*Fa*PAL); (**B**) Chalcone synthase (*Fa*CHS); (**C**) 3-deoxy-D-arabinoheptulosonate 7-phosphate Synthase (*Fa*DAHPS); (**D**) 3-dehydroquinate Synthase (*Fa*DHQS); (**E**) 3-dehydroshikimate Synthase (*Fa*DHD-SDH2) and (**F**) 5-enolpyruvylshikimate 3-phosphate Synthase (*Fa*EPSPS). Each bar represent the result of three technical replicates from five experimental samples (n = 5) ± SE. In each test different letters indicate a significant difference among treatments and controls (Duncan’s test, p ≤ 0.05, p ≤ 0.10).

**Figure 4:**
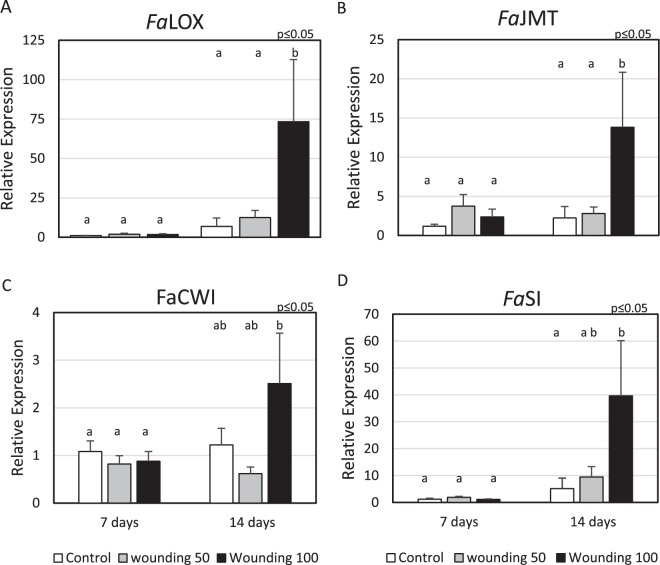
Relative expression of sugar transport involved genes. Application of two levels of preharvest wounding on leaves and its effects in strawberry fruit when wounding occurred 7 and 14 days before harvest. (**A**) Lipoxygenase (*Fa*LOX); (**B**) Jasmonic acid carboxyl methyltransferase (*Fa*JMT); (**C**) Cell wall invertase (*Fa*CWI) and (**D**) Soluble invertase (*Fa*SI). Each bar represent the result of three technical replicates from five experimental samples (n = 5) ± SE. In each test different letters indicate a significant difference among treatments and controls (Duncan’s test, p ≤ 0.05, p ≤ 0.10).

Phenylalanine ammonia lyase (PAL) and chalcone synthase (CHS) are important enzymes involved in polyphenol biosynthesis^22^. PAL is the first and limiting step in the phenylpropanoid pathway and CHS is the first committed enzyme in flavonoid biosynthesis^23^. When wounding occurred two weeks before harvest, for W100 treated plants, PAL increased 1.85 fold and CHS 1.73 fold (Fig. 3A,B). The expression of genes encoding 3-deoxy-D-arabino-heptulosonate 7-phosphate synthase gene (*Fa*DAHPS) and 3-dehydroquinate synthase (*Fa*DHQS) were not affected by the application of wounding 7 or 14 days before harvest (Fig. 3C,D). DAHPS is the first enzyme in the shikimate pathway and catalyze the reaction of phosphoenolpyruvate with D-erythrose 4-phosphate to produce 3-Deoxy-D-arabinoheptulosonate 7-phosphate (DAHP) and releasing phosphate. DHQS catalyzes the second step in the shikimate pathway using DAHP as a substrate to produce 3-dehydroquinate and phosphate^23^. The relative expression of 3-dehydroshikimate Synthase (*Fa*DHD-SDH2) implicated in the synthesis of gallic acid from shikimic acid^24^ was 15.2-fold over the control for the higher wounding level applied 14 days before harvest (Fig. 3E). *Fa*EPSPS 5-enolpyruvylshikimate-3-phosphate synthase gene expression (Fig. 3F) was also greater in fruits from wounded plant 14 days before harvest (6.25 fold). This enzyme catalyzes the reaction that transforms shikimate-3-phosphate and phosphoenolpyruvate to 5-enolpyruvylshikimate-3-phosphate (EPSP). Lipoxygenase (LOX) is an enzyme involved in the first steps of the biosynthesis of JA, catalyzing the oxidation of alpha-linolenic acid, producing linolenic acid hydroperoxide^25^. Figure 4A shows a 10.7 fold increment in the transcript for the LOX gene in strawberry fruit from wounded plants (W100) applied 14 days before harvest. The expression of jasmonate methyl transferase (JMT), which catalyzes the conversion of JA to MeJA by adding a methyl group^26^, increased 6.2 fold in fruit when wounding was applied on leaves 14 days before harvest (Fig. 4B). The expression of sucrose invertases genes also increased; 2 fold for cell wall invertase (CWI, Fig. 4C) and 7.7 fold for soluble invertase (SI, Fig. 4D).

Table 2 shows the average losses in the foliar area for the wounding treatments, resulting in approx. 2.50% for W100 and 1.25% for W50 treated plants.

**Table 2:** Estimated averages area losses for leaflet in strawberry plants.

	Area (cm^2^)	Area loss of leaves due to wounding (%)
Leaflet non-wounded (one leaf)	30.81	
Leaflet wounded (5 holes)	29.46	
Wounded area of 5 holes	1.35	
Total foliar area of 35 leaves/plant	1078.35	—
Wounding area (50 holes/plant)	13.50	1.25
Wounding area (100 holes/plant)	27.00	2.50

## Discussion

Organic agriculture claims that under this kind of management fruits produce more phytochemicals than under the conventional approach^3,4^. This claim is supported by several reports comparing both systems^27–29^; alternatively, many studies indicate there are no differences^30–32^, setting a controversial matter for several years. There is a speculation that biotic stress due to insect and pathogen attacks triggers the production of defensive secondary compounds, but this hypothesis has never been tested before. The wounding component of the biotic stress is already known as a cause of the overproduction of secondary metabolites, mainly phenolic compounds that accumulate in the damaged leaf and also in other distant leaves as a systemic defensive response^33,34^. The relation between invertase activity and carbon transport is important for the modulation of plant defense and secondary metabolism as carbon is the source for phenolic compounds and sucrose and glucose play roles as signaling molecules^35,36^. There is evidence which suggest that translocation of sucrose from source tissues to distant sink tissues increases the production of defensive molecules^37,38^. Invertases may be triggered as a local or systemic respond to wounding. For instance in pea, the cell wall invertase (CWI) was overexpressed by application of wounding and jasmonic acid in the same leaves, but the systemic induction in other leaves was not detected^39^. On the other hand, systemic induction of CWI by wounding was observed in *Populus* sp. in a source-sink model involving different leaves^40,41^. Since it is known that invertases bounded to plant cell walls facilitate phloem unloading at fruit tissues by hydrolyzing sucrose into glucose and fructose, we hypothesized that wounding stress on leaves could trigger a systemic response of invertases in fruit tissue.

**Figure 5:**
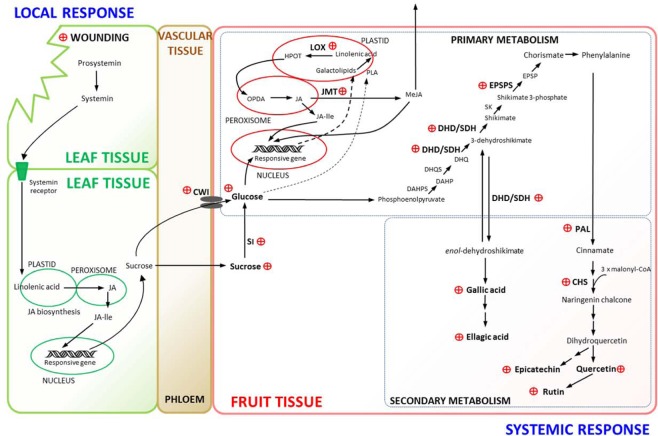
Proposed model for phenolic biosynthesis in strawberry fruit induced by long distance wounding applied on leaves before harvest. Leaf wounding triggers systemin signaling and a JA/JA-Ile mechanism as a local response, reconfigures the sugar metabolism, sucrose upload in the vascular tissue and its transport to the fruit. Systemic response in fruit shows upregulation of sucrose invertases genes (CWI, SI), increasing soluble sugars and defensive genes transcripts related to phenolic compounds biosynthesis (LOX, JMT, DHD/SDH, EPSPS, PAL, CHS) and the corresponding phenylpropanoids (quercetin, rutin and epichatechin) and hydrolysable tannin derivatives (ellagic acid and gallic acid). JA, Jasmonic acid; JA-Ile, Jasmonic acid isoleucine; LOX, Lipoxygenase; JMT, Jasmonate methyl transferase; OPDA, 12-oxo-phytodienoic acid; HPOT, 9-/13-hydroperoxy-octadecatrienoic acid; CWI, Cell wall invertase; SI, Soluble invertase; PAL, Phenylalanine ammonia lyase; CHS, Chalcone synthase; DHD/SDH, 3-dehydroshikimate synthase; EPSPS, 5-enolpyruvylshikimate 3-phosphate synthase. Applied wounding stress and up regulation of enzyme genes and associated primary and secondary metabolites increments are represented by a red ⊕ symbol.

Based on the results found in the present study and on previous research, we propose a model explaining the possible mechanism of polyphenol biosynthesis in strawberries as a result of distant wounding stress exerted in the plant leaves (Fig. 5). Wounding stress applied on leaves days before harvesting the strawberry fruit, upregulates systemic gene expressions associated to carbon partition, MJ biosynthesis and polyphenol biosynthesis in the fruit. The qRT-PCR analyses detected the overexpression of genes implied in the phenolics pathway including Phenylalanine ammonia lyase (*Fa*PAL); Chalcone synthase (*Fa*CHS); 3-dehydroshikimate Synthase (*Fa*DHD-SDH2) and 5-enolpyruvylshikimate 3-phosphate Synthase (*Fa*EPSPS). The carbohydrate metabolism on the strawberry fruit was also affected, where total sugar content increased as a late response of wounding induced on leaves and confirmed at molecular level with higher expression of sugar transporter genes (CWI and SI). Moreover, the expression of defense responsive genes was also affected in fruit (LOX and JMT). In this model, the wounding produced on the leaves triggers the local response through releasing systemin from prosystemin in wounded tissue which binds to systemin receptors at intact tissue membranes^42^. At the plastid level the JA/JA-Ile mechanism is triggered and the responsive genes activate the defensive mechanism^42,43^. That reconfigures the sugar metabolism, producing an upload of sucrose in the vascular tissue that is transported to the fruit. In fruits, the up-regulation of sucrose invertases genes (CWI and SI), allow the increase of soluble sugar in the cells. The imbalance in sucrose/glucose triggers the octadecanoic pathway^14,44^ increasing the JMT transcripts and defensive genes related to phenolic compounds biosynthesis. The greater accumulation of soluble sugar in fruit cells also increases the availability of carbon for the biosynthesis of secondary metabolites with high C/N ratio^45^, such as the phenylpropanoids (quercetin, rutin and epicatechin) and hydrolysable tannin derivatives (ellagic acid and gallic acid) as shown in this study. *In situ* wounding effects on phenolic biosynthesis are ROS mediated^12^ while in the present study ROS levels did not increase within the fruit supporting the idea that wounding systemic effects on phenolic accumulation are ROS-independent.

## Conclusions

Here is reported for the first time the accumulation of phenolic compounds in fruit through long distance wounding applied to leaves, in an experiment conducted with strawberry plant in field conditions. The results clarify the role of wounding for the accumulation of defensive compounds in fruit. The results support the idea that higher levels of phytochemicals reported in organic fruits and vegetables could be due to the wounding component of the biotic stress attributed to herbivore insects feeding on leaves, to which the plant is exposed. The delayed response of fruits in synthesizing phenolics is the result for a late defensive response. This produced an accumulation of soluble sugars in fruits as source of carbon for high rate C/N secondary metabolites production mediated by a MJ mechanism. In general, this reported systemic preharvest long distance wounding response on phenolic biosynthesis in fruit is carbon partition and MJ-mediated and differs from previous reports of local postharvest wounding response of phenolic biosynthesis which is mainly ROS-mediated^46^.

As a technological application of the present results, the controlled mechanical wounding applied during preharvest in leaves could be used to increase phytochemicals in fruit. Further studies are recommended in other crops as well.

## Materials and Methods

### Field experiment

The field experiment was conducted on a strawberry (*Fragaria* × *ananassa*) plots at the end of the harvesting season, from December 2013 to January 2014, at INIA-Las Brujas Research Station, Uruguay (lat. 34.66 S, long. 56.34 W). An evaluation plot of the advanced clone selection LBM 10.3 (cv. Albion X SGG 31.1) was used for the experiment (Fig. 1A). Each treatment was a plot in a brunisol soil. The use of insecticides or fungicides was not necessary because there was neither incidence of *Botrytis* sp. nor noticeable presence of insects. Inside each plot, 20 plants (n = 20) were assigned to each treatment as follows:

(1) Control with no perforations applied to the plants; (2) Low mechanical wounding (W50) consisted of 3–5 perforations per leaf (Fig. 1B), for a total of 50 perforations applied randomly to 10 leaves per plant; (3) High mechanical wounding with 100 perforation on each plant (W100), applied randomly to 20 leaves per plant. Perforations were made with a manual paper puncher 7 or 14 days before harvesting (3 months after flowering).

The wounding was applied to plants with fully developed fruits (25% red color). Each plant was harvested one and two weeks after the treatment was applied, selecting the full ripened fruits (over 80% of full color).

The wounding stress was applied without affecting the foliar area beyond 2.5% (Table 2), to not alter the photosynthetic activity of the leaves and the rate of water loss based on previous studies^47^, where a 10% leave damage by insects caused a 25% reduction in photosynthesis and 25% decrease in water loss during day time and a 34% increase in water loss in night-time in sycamore trees. The selected wounded area in the present study could mimic a foliar attack by insects without affecting the normal performance of the plant.

### Harvest evaluation

Immediately after harvest, fruits were evaluated for harvest quality by measuring physicochemical parameters. For each fruit, the weight was registered and color was determined by two measures using a digital colorimeter (CR-200, D65 illuminant; Minolta, Tokyo, Japan), recording the L*a*b* coordinates values. Fruit firmness was determined using a TA.XTPlus Texture Analyzer (Stable Mycro System, Surrey, United Kingdom). The soluble solids concentration (SS) was determined in the juice by an Atago RX-1000 digital refractometer (Atago Co. Ltd, Tokyo, Japan). After these determinations, samples were frozen in liquid nitrogen and stored at −80 °C for subsequent freeze drying and further analyses. All samples were freeze-dried in a FreeZone benchtop freeze dry system (Labconco, Kansas City, MO) until they were completely dried.

### Total phenolics and vitamin C analysis

Total phenolic compounds and total vitamin C were determined in the same analysis according to the method reported by Sanchez-Rangel, *et al*.^48^. Briefly, 50 mg of freeze dried strawberry powder was extracted with 1 mL of MeOH:H_2_O (80:20 v/v) in a centrifuge tube using an ultrasonic bath for 30 min. Samples were then centrifuged at 14,000 rpm. In a 96 plate were added 15 µl of extract and 240 µl of distilled water followed by 15 µl of Folin-Ciocalteau reagent. The mixture was incubated for 3 min and the absorbance was read at 725 nm for estimation of vitamin C. After that a Na_2_CO_3_ solution (30 µl, 1 N) was added and incubated at room temperature in dark conditions for 2 h, and then the absorbance was measured again at 725 nm. In parallel, standards of ascorbic and chlorogenic acid were run in addition of blanks. Absorbance was recorded using a Synergy-HT Microplate Reader and analyzed using KC4 software (Bio-Tek Inc., Winooski, VT).

### HPLC analysis of phenolic compounds

The identification of individual compounds were performed in a LCQ Deca XP Max LC-MS^n^ system (Thermo Finnigan, CA) equipped with an autosampler, a Surveyor 2000 quaternary pump and a UV 2000 PDA detector, using a 150 × 2.00 mm Synergi 4 µ Hydro RP 80 A column (Phenomenex, Torrance, CA) and a guard column of the same chemistry. The LC-MS^n^ system with a *Z*-spray ESI source was run by Xcalibur software, version 1.3 (Thermo Finnigan-Surveyor, San Jose, CA, USA). The mobile phase flow rate was set at 0.25 mL/min, while the elution gradients were performed with solvent A, consisting of acetonitrile/methanol (1:1) (containing 0.5% formic acid); and solvent B, consisting of water (containing 0.5% formic acid). The applied elution conditions were: 0–2 min, 2% A, 98% B; 3–5 min, 5% A, 95% B; 5–7 min, 25% A, 75% B; 7–12 min, 55% A, 45% B; 12–24 min, 55%A-80%A, 24–27 min held isocratic at 80%A, 28–30 min 90% A, 10% B; 31–33 min held isocratic, 100% A; 34–40 min, 2% A, 98% B, to the starting condition. The chromatograms were monitored at 280 nm, and complete spectral data were recorded in the range 200–600 nm. ESI was performed in the negative ionization mode, nitrogen was used as sheath gas with a flow of 59 arbitrary units, and He gas was used as dampening gas. The capillary voltage, −4.17 V; spray voltage, 5 kV; capillary temperature, 275 °C; and tube lens voltage at −55V. Collision energies of 30% were used for the MS^n^ analysis.

Chromatographic separation and quantification was performed on same LC-MS^n^ system. The elution gradient was formed with solvent A [0.5% formic acid -water] and solvent B [0.5% formic acid in acetonitrile]. A linear gradient was set up with A and B: 0 min 98% A, 10 min 75% A, 20 min 75% A, 30 min 25% A, 35 min 0% A, 38 min 98% A. The flow rate was 200 µl/min. The injection volume was 10 µl. Retention time and spectral profile were used for identification detected by a photodiode array detector (PDA) scanning between 190–600 nm, the quantification was done by comparison with external standards obtained from Sigma-Aldrich (St. Luis, MO).

### Total ROS measurement

The ROS measurement from strawberry fruits were carried out by using 2ʹ,7ʹ-Dichlorofluorescein diacetate^49^ (DCFDA) (Sigma, St. Louis, MO). Briefly, strawberry fruit samples were ground into fine powder under liquid nitrogen and then ~10 mg of fine powders were mixed with 350 μl of 10 mM Tris–HCl (pH 7.2) and then centrifuged at 12,000 × g for 20 min at 4 °C. The supernatant (~300 μl) was transferred to a fresh 1.5 ml tube and further subjected to ROS measurement using the black 96-well black clear-bottom plate (Costar, Cambridge, MA). Each well included 200 μl of total reaction mixture, composed of 50 μl of the supernatant and 150 μl of 10 mM Tris–HCl (pH 7.2) including 13.3 μM DCFDA. After incubation at room temperature in dark for 20 min, fluorescence was read immediately at wavelengths of 485 nm for excitation and 528 nm for emission on a 96-well microplate reader (Synergy HT, Bio-Tek Instruments, Inc., Winooski, VT). The 10 mM Tris–HCl (pH 7.2) was used as a blank and the relative ROS level of each sample was normalized by exact amount (mg) of each sample used for the ROS measurement. Finally, data was obtained from five biological repeats with two technical repeats.

### Gene expression

Total RNA extraction from strawberry fruits was carried out by combining the method previously described by Christou, *et al*.^50^, with the RNeasy Plant Mini Kit (Qiagen, Valencia, CA). Briefly, 0.1 g of freeze-dried strawberry fruits was mixed with 1 ml of the extraction buffer (0.5 M Tris–HCl pH 8.8 and 1% sodium dodecyl sulfate [SDS]). Subsequently, 1 ml of phenol:chloroform:isoamyl alcohol (PCI) (25:24:1 [v/v]) was added to the mixture, which was gently agitated and then centrifuged at 14,000 rpm for 5 min at 4 °C for phase separation. The upper aqueous phase (~800 μl) was further subjected to the PCI extractions (three times). After the third PCI extraction, the upper aqueous phase (~400 μl), whose phenol traces were removed completely, was collected into a fresh chilled tube, where 0.1 volume of 3 M NaOAC (pH 5.6) and 1 volume of 100% ethanol were mixed, incubated at −80 °C for 20 min and then centrifuged at 12,000 rpm for 8 min at 4 °C for RNA precipitation. After drying at room temperature, RNA pellets were finally dissolved in RNase-free water and further purified by RNeasy Plant Mini Kit (Qiagen, Valencia, CA) according to the manufacturer’s instructions. RNA concentration was measured with a NanoDrop ND-1000 spectrophotometer (NanoDrop Technologies, Willmington, DE). Aliquots of 0.7 µg RNA, treated with DNase I to avoid DNA contamination, were reverse-transcribed into cDNA using the SuperScript III first-strand synthesis supermix (Invitrogen, Carlsbad, CA) following the manufacturers protocol. Finally, the cDNAs were used for real-time qRT-PCR analyses, which were performed using Power SYBR Green PCR Master Mix (Applied Biosystems, Foster City, CA), following the manufacturer’s instructions. cDNA amplification was carried out using a 7900 HT Sequence Detection System (Applied Biosystems, Foster City, CA). The primer sets used in this study were provided by Integrated DNA Technologies (IDT, Coralville, IA), and their sequence information is shown on Table 3. The relative expression of each gene was normalized by the *FaGAPDH* and was calculated following the comparative Ct method (ΔΔCt), known as the 2^−ΔΔCt^ method. Strawberry gene were selected based on implication in the shikimate pathway, phenolic compounds biosynthesis, and sugar transport and metabolism. All of these genes have been reported for strawberry fruits, and primer sequences are available^51,52^.

**Table 3:** Sequence of primers from *Fragaria* *x ananassa* used in qRT-PCR analyses.

Primer	Sequence
*FaPAL-F*	5′-CACCTGCTCTCAGTCGTGGACC-3′
*FaPAL-R*	5′-GCA TGTTCTACTAGCTCTGCCCTCAG-3′
*FaCHS-F*	5′-GTTGGGCTCACATTTCACCTCCTCA-3′
*FaCHS-R*	5′-AATTGCTGGGCCACCTGGGTG-3′
*FaEPSPS-F*	5′-GGAGACTTGGTCACTGGTCTTA-3′
*FaEPSPS-R*	5′-GAAGGCCTCCCTTTCCAATTAC-3′
*FaDAHPS-F*	5′-CGCAACTGGTGGGTATGCGGC-3′
*FaDAHPS-R*	5′-CCCGGTGAGCAAGTTCCCGG-3′
*FaDHQS-F*	5′-GCAGCTGGCATGATCATGGCTG-3′
*FaDHQS-R*	5′-CGGTCACAGACTCAGGAGGGC-3′
*FaDHD/SDH 1-F*	5′-AGCTCCTGGTCAACCTACTATC-3′
*FaDHD/SDH 1-R*	5′-GCTGACGGGCTTTCCAATAA-3′
*FaDHD/SDH 2-F*	5′-CGTTGGGATTCCTCACAAAGA-3′
*FaDHD/SDH 2-R*	5′-CATCAGTTGGCCTCCTTACAA-3′
*FaDHD/SDH 3-F*	5′-GAGGAAGGACTTCGAGGATTAG-3′
*FaDHD/SDH 3-R*	5′-GCTCCCATGACCACAAATAAC-3′
*FaSI-F*	5′-GGTATGTGGGAGTGCATTGA-3′
*FaSI-R*	5′-CGTCCAAGCTAGCCTTTAGAA-3′
*FaCWI-F*	5′-CCAGGCAATTCCAAGGACTAT-3′
*FaCWI-R*	5′-CTTGACCTCGTTTGTTCTAAGTTT C-3′
*FaLOX-F*	5′-CCGGGACACGATGAACATAA-3′
*FaLOX-R*	5′-GGCATATTGAGCTGGGAAGA-3′
*FaJMT-F*	5′-AATAAGCAGCGGCGAGCGAGTAGC-3′
*FaJMT-R*	5′-AAGCGATCACTGACGAGCTCTGCG-3′
*FaGAPDH-F*	5′-TCCATCACTGCCACCCAGAAGACTG-3′
*FaGAPDH-R*	5′-AGCAGGCAGAACCTTTCCGACAG-3′

The sequences belong to genes of enzymes involved in phenolics biosynthesis and other related enzymes.

### Foliar area

Estimation of losses of foliar area for each strawberry plant was done using the software ImageJ. Two average leaflets were picked, submitted to wounding and a digital picture was taken. Twelve leaves were considered the average amount of leaves for this advanced selection.

### Statistical analysis

Analysis of Variance (ANOVA) was applied to the data and the statistical differences between treatment means were determined using the Duncan’s Test (p ≤ 0.05 and p ≤ 0.1). Those tests were conducted using the software Statistica 9.0 (StatSoft, Tulsa, OK).
